# Alternol/Alteronol: Potent Anti-cancer Compounds With Multiple Mechanistic Actions

**DOI:** 10.3389/fonc.2020.568110

**Published:** 2020-11-03

**Authors:** Wang Liu, Jean C. Li, Jian Huang, Jiepeng Chen, Jeffrey Holzbeierlein, Benyi Li

**Affiliations:** ^1^Department of Urology, The University of Kansas Medical Center, Kansas City, KS, United States; ^2^Institute of Molecular Pathology, The Affiliated Hospital, Guangdong Medical University, Zhanjiang, China; ^3^Sungen Bioscience Institute, Shantou, China

**Keywords:** Alternol, Alteronol, apoptosis, cell cycle, radical oxygen species, Cladosporol

## Abstract

Alternol and its oxidate isomer Alteronol are small compounds isolated from the fermentation of a mutant fungus obtained from *Taxus brevifolia* bark. Preclinical studies showed their potent anti-cancer activities, including attenuating cellular survival pathways, altering protein levels of cell cycle regulators, activating xanthine dehydrogenase to cause accumulation of cellular reactive oxygen species and disrupting cell metabolism by disturbing four Krebs cycle enzymes specifically in malignant cells while having no significant effect on benign cells. In cancer cell culture models, Alternol or Alteronol exert their anti-cancer effect by inducing cell cycle arrest and triggering apoptotic cell death. In mice xenograft models, Alternol or Alteronol potently suppresses tumor growth with no obvious toxicity to the host with a wide therapeutic index over 30-fold. In conclusion, Alternol or Alteronol possess a great potential and feasibility to be developed as an effective anti-tumor therapeutic.

## Introduction

**FIGURE 1:**
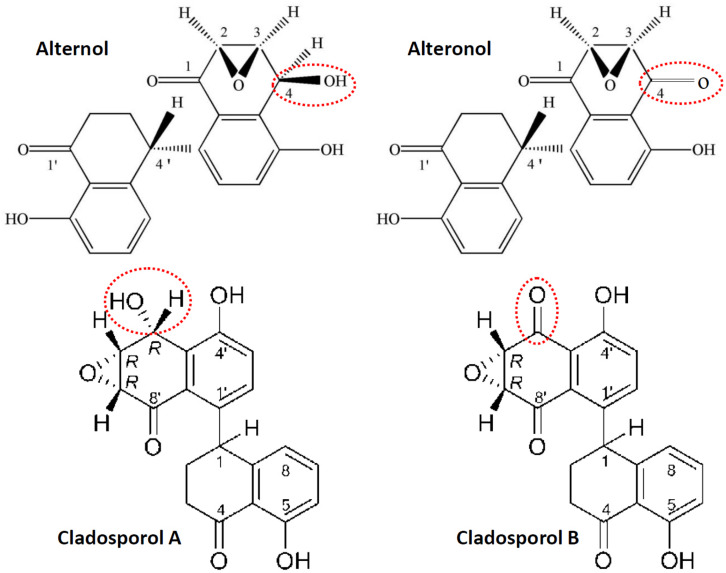
Chemical structures for Alternol, Alteronol and Cladosporols. The differences between these two pairs of isomer compounds were highlighted in red-dotted circle, indicating the oxidate site in Alteronol and Cladosporol B.

Alternol (Formula C_20_H_16_O_6_, MW 352.3) and Alteronol (Formula C_20_H_14_O_6_, MW 350.3) were purified from the fermented extracts of a mutant fungus *Alternaria alternate var.monosporus* (1). The wild-type of this fungus was isolated from the bark of *Taxus brevifolia*, the same source for paclitaxel purification (2) and was then mutagenized *via* UV irradiation to produce high quantity of paclitaxel (3, 4). A dimeric binaphthyl chemical (named as Alterfungin) was highly yielded from the mutant fungal extract (5). Chemical structure analysis revealed that Alterfungin is a chiral isomer of Cladosporol (6), a secondary metabolite originally isolated from a hyperparasite of rust fungi *Cladosporium tenuissimum* (Figure 1). Interestingly, Cladosporol was also purified late on from a fermentation broth of the paclitaxel-producing fungus *Alternaria alternate var.monosporus* (7) and exhibited a moderate anti-cancer effect on multiple human cancer cell lines *in vitro* and *in vivo* (7–11). Later on, Alterfungin was re-named as Alternol (12) and its oxidate derivative was named as Alteronol (13). As the isomers, Alternol and Alteronol share the same physical and chemical properties with only one difference at the position 4 due to oxidation; the hydroxyl group on Alternol is replaced by a carbonyl group in Alteronol (Figure 1). In the past 13 years, Alternol and Alteronol have been tested in a variety of cancer cell lines *in vitro* and animal xenograft models *in vivo* for their anti-cancer potential (Table 1). Accumulating data demonstrated that these compounds selectivity inhibit cancer cell proliferation *in vitro* and suppress tumor growth *in vivo* without obvious toxicity to benign cells or host animals. This review summarizes the research findings of their anti-cancer effects and underlying mechanisms.

**TABLE 1:** Summary of current studies for Alternol/Alteronol effect *in vitro* and *in vivo.*

Organ/tissue	Cell line	Cell biological effect	Molecular mechanism	Anti-tumor effect *in vivo*	References
Prostate cancer	PC-3, 22RV1, C4-2, LNCaP, DU145	ROS accumulation, cell protein oxidative increase	Increase the expression and activity of XDH protein		(30)
	PC-3, 22RV1, C4-2	Mitochondrial respiration and ATP production attenuation	Inhibit the activity of PDH/KGDH complex	Inhibit ATP production in PC-3 xenograft model	(37)
	LNCaP, C4-2, 22RV1, PC-3, DU145	ROS accumulation, cell apoptosis, mitochondrial dysfunction	Activate Casp-3 and Bax, decrease Bif-1, decrease the ratio of Bcl-2/Bcl-XL	Inhibit the growth of PC-3 xenograft model	(18)
	C4-2, RWPE-1	Autophagy defense response	Activate AMPK protein, increase the phosphorylation of p27		(17)
Melanoma	UACC62, A375, WM35	Inhibit cell proliferation, lead to apoptosis and autophagy	Decrease the phosphorylation of AKT/mTOR protein		(26)
	B16F1	Cell proliferation and migration inhibition	Decrease the level of MMP2 protein	Inhibit B16F1 tumor metastasis *in vivo*	(26)
	B16F0	Cell cycle arrest in S phase	Increase the ratio of Bax/Bcl-2, activate Caspase-3/9 protein		(27)
	B16F0	Inhibit cell proliferation and lead to cell differentiation	Increase the expression of melanin	Delay B16F0 tumor growth *in vivo*	(34)
Lung cancer	A549	Inhibit cell proliferation and lead to apoptosis	Activate Caspase-3/9 protein		(25)
Cervical cancer	U14, HeLa	Inhibit cell proliferation and lead to apoptosis	Decrease the expression of Bcl-2/Survivin, increase the expression of Bax		(28)
		Cell cycle arrest in G1 phase	Down-regulate the expression of Cylcin D1 protein		(13)
Lymphoma	L210	Inhibit cell proliferation and lead to apoptosis	Decrease the ratio of Bcl-2/Bax		(12, 16)
		Mitochondrial transmembrane potential (ΔΨm) depolarization, ROS accumulation			
Hepatoma	HepG2	Inhibit cell proliferation, EMT and lead to apoptosis			(19)
		Cell cycle arrest in G2/M phase			
Breast cancer	4T1, MCF7	Inhibit cell proliferation and lead to apoptosis	Down-regulate the expression of Cylcin B1 protein	Inhibit the growth of 4T1 xenograft model	(23, 24)
		Cell cycle arrest in G2/M phase	Activate Casp-9/Casp-3/PARP apoptosis pathway		
		ROS accumulation	Activate JNK/p38 signaling pathway		
Gastric cancer	MGC803	Inhibit cell proliferation and lead to apoptosis	Increase the expression of CDC2/pY15, decrease the expression of PLK1 protein		(15)
		Cell cycle arrest in G2/M phase	Increase the expression of p53/p21		(15)
Esophagus cancer	ECA-109	Inhibit cell proliferation and lead to apoptosis			(15)
Ovarian cancer	A2780	Inhibit cell proliferation and lead to apoptosis			(15)
Pancreatic cancer	PANC-1, BxPC3	Inhibit cell proliferation and lead to apoptosis	Activate Caspase-3, increase the expression of p53/p21, decrease the expression of Bcl-2		(21)
		Cell cycle arrest in S phase			
Osteosarcoma	143B, KRIB, MG63, U20S	Inihibit cell proliferation and migration, lead to cell apoptosis	increase the expression of p21/p27/cyclin B1, decrease CDC2 protein level	Inhibit the growth of 143B xenograft model	(22)
		Cell cycle arrest in G2/M phase	Activate Casp-8/Casp-3/PARP apoptosis pathway		
			Activate MAPK/JNK/p38 kinases and inhibit STAT3 activity		
Lymphoblastoma	HL60	Inhibit cell proliferation	Down-regulate the expression of Cyclin D1 and Rb protein		(20, 27)

*AMPK, AMP-activated protein kinase; CDC2, cell division cycle protein 2; Casp-3/9, caspase-3/9; KGDH, α-ketoglutarate dehydrogenase; MMP, matrix metalloproteinase; mTOR, mammalian target of rapamycin; PDH, pyruvate dehydrogenase; PLK1, polo-like kinase 1; ROS, reactive oxygen species; STAT3, Signal transducer and activator of transcription 3; XDH, xanthine dehydrogenase.*

## Inhibition of Cancer Cell Proliferation

Malignant tumor growth is always accompanied with rapid cell proliferation, inactivation of cell cycle checkpoints and aberrant expression of cyclin proteins (14). To investigate its anti-cancer effect, Alternol was first applied to human gastric cancer cell MGC-803 and murine leukemia L1210 cells *in vitro*, and the results showed a prominent inhibition of cell proliferation (15, 16). These anti-cancer effects by Alternol and Alteronol were extended to a variety of human cancer types, including prostate (17, 18), liver (19), cervical (13), leukemia (20), pancreatic (21), osteosarcoma (22), breast (23, 24), lung (25) and melanoma (26). Dose-response experiments determined that the concentration range of 50% growth inhibition (GI_50_) was between 2 and 10 μM at 24–48 h treatment (12, 13, 15–24, 26, 27). However, lung cancer A549 cells were not sensitive to Alternol (GI_50_ at 37 μM) (25) compared to other cell lines and prostate cancer DU145 cells are resistant to Alternol due to lack of Bax protein expression (18).

**FIGURE 2:**
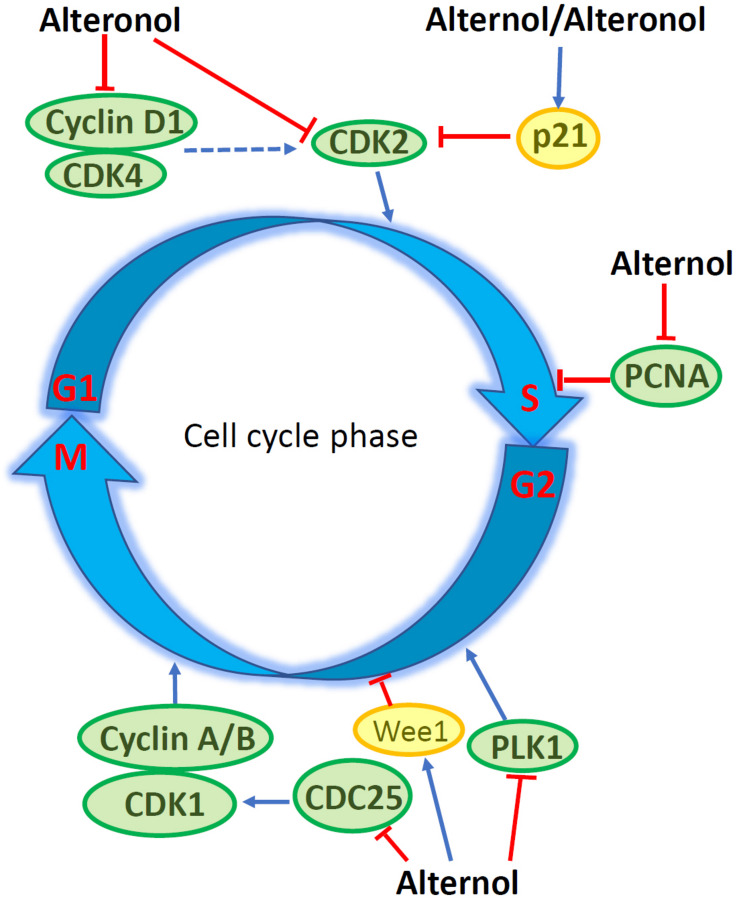
Alternol/Alteronol induce cell cycle arrest in cancer cells by attenuating the expression of cell cycle regulators, including down-regulation of CDK2/4, Cyclin D1, PCNA, PLK1 and CDC25 expression and up-regulation of Wee1 and p21^*cip*1/waf1^ expression.

Flow cytometry analysis revealed that Alternol induced G_2_/M cell cycle arrest, which was related to a significant reduction of polo-like kinase 1 (PLK1) protein, a major regulator of G_2_/M transition, in parallel with reduced CDC25C and elevated Wee1 protein levels (15). In murine melanoma B16F0 and B16F10 cells, Alternol induced S phase cell cycle arrest, but had a lesser effect on human embryonic kidney 293T cells (27). Similar S phase arrest was also reported in human pancreatic cancer PANC-1 and BxPC3 cells (21). Further analysis determined that Alternol treatment increased CDK inhibitory protein p21^*cip*1/waf1^ expression and reduced the expression of proliferating cell nuclear antigen (PCNA) and cyclin-dependent kinase 2 (CDK2) proteins, leading to S phase cell cycle arrest (27). In human cervical cancer HeLa cells, Alteronol inhibited cell proliferation by causing G_1_ phase cell cycle arrest, which was associated with reduced expression of CDK2, CDK4, cyclin D1 and an increased p21^*cip*1/waf1^ expression (13). Interestingly, Cladosporol also caused a similar effect on G_1_ phase arrest and increased p21^*cip*1/waf1^ gene expression in multiple human colon cancer cells upon activating PPARγ-related pathway (8–10). However, in human breast T47D and 4T1 cells, Alteronol inhibited cell proliferation *via* G_2_ phase arrest, possibly due to increased p21^*cip*1/waf1^ expression and decreased expression of CDC2 and cyclin B1 (23, 24). Also, a significant synergistic effect of Alteronol plus Adriamycin was observed in murine breast cancer 4T1 cell (24). These studies indicate that Alternol and Alteronol inhibit cancer cell proliferation by inducing cell cycle arrest *via* cell-specific mechanisms (Figure 2 and Table 1).

## Induction of Apoptotic Cell Death

**FIGURE 3:**
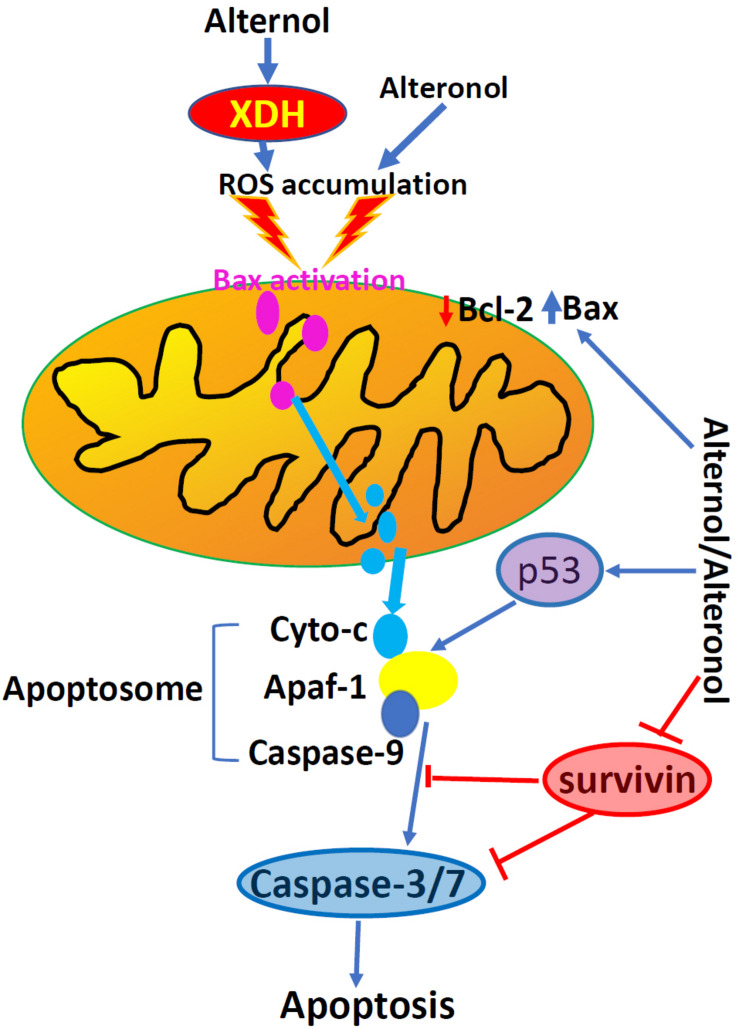
Alternol and Alteronol induce ROS accumulation by activating cytosolic XDH, resulting in Bax activation, cytochrome c release and apoptosis. Alternol/Alteronol also reduces the expression of anti-apoptotic Bcl-2 and Survivin proteins but enhances the expression of pro-apoptotic p53 protein, leading to intrinsic apoptotic cell death. Pink dots denote activated Bax proteins and light-blue dots denote cytochrome c proteins.

Apoptosis is one of many forms of programmed cell death and dysregulation of apoptosis is one of the basic characteristics in malignant tumors (14). Alternol-induced apoptosis was first reported in L1210 and MGC803 cells, accompanied with cellular reactive oxygen species (ROS) accumulation and a reduction of Bcl-2/Bax ratio (12, 15, 16). This apoptotic effect was later confirmed in many other cancer cell types (18, 21–24), except prostate cancer DU145 and leukemia HL60 cells, which showed apoptotic resistance (18, 20). Alternol- or Alteronol-induced apoptosis is mechanistically caused by the disturbance of pro- and anti-apoptotic Bcl-2 family proteins and the damage of mitochondrial membrane potential, leading to cytochrome c release and caspase activation (18, 21, 23, 24). In addition, Alternol reduced survivin expression in parallel to Bcl-2 reduction in murine cervical cancer U14 cells (28), and Alteronol increased p53 expression in breast cancer cells (23). Most importantly, Alternol selectively induced apoptosis in prostate cancer LNCaP, C4-2, PC-3 and 22RV1 cells in a time- and dose-dependent manner, without a significant effect on benign prostatic RWPE-1 and BPH1 cells (18). In depth analysis revealed that ROS-dependent Bax protein activation is a major mechanism in Alternol-induced apoptosis in prostate cancer cells (18). These data indicate that Alternol or Alteronol triggers an intrinsic apoptotic pathway to induce cancer cell death but sparing benign cells (Figure 3 and Table 1).

Owing to rapid cell growth, cancer cells generate excessive ROS compared to benign cells. This has been used as a therapeutic hit for anti-cancer drug development (29). Although Alternol-induced ROS accumulation was first reported in L1210 cells (12), and the causative role of ROS accumulation in Alternol-induced apoptosis was only demonstrated later in prostate cancer cells (18). Alternol-induced ROS accumulation peaked at 4 h after drug treatment, and Alternol-induced apoptosis was abrogated by ROS scavengers *N*-acetylcysteine (*N*-Ac) and dihydrolipoic acid, which was supported by studies from different groups (22, 23). A similar effect was also reported using Alteronol and Cladosporol in breast cancer cells (11, 23, 24).

The major source of cellular ROS accumulation induced by Alternol treatment was recently defined as the cytoplasmic xanthine dehydrogenase (XDH, also called xanthine oxidase, XO) using pharmacological inhibitors and genetic approaches (30). In prostate cancer cells, Alternol treatment only moderately increased mitochondrial superoxide formation that was significantly lower than the total cellular ROS level, indicating that the mitochondrial ROS source was not the major contributor. Also, total cellular ROS level or cell death after Alternol treatment was not reduced by pre-treatment with mitochondria-specific antioxidant MitoQ, inhibitors for NADPH oxidase (NOX) or nitric oxide synthase (NOS). Conversely, XDH-specific small chemical inhibitors or gene silencing reduced total cellular ROS levels and protected cells from apoptosis induced by Alternol. Further analysis revealed that Alternol treatment significantly enhanced XDH oxidative activity and induced a profound cellular protein oxidation in malignant but not in benign cells. Meanwhile, the study also discovered that benign cells had a dramatic increase of antioxidant superoxide dismutase (SOD) and catalase activities compared to malignant cells after Alternol treatment, indicating a potential mechanism for Alternol’s low toxicity to benign cells. In addition, an *in-silicon* docking analysis suggested that Alternol interacts with the XDH protein at two amino acid residues K755 and R787 within its catalytic molybdenum binding domain (30). Therefore, Alternol is considered as an XDH agonist, leading to excessive ROS generation, cellular stress and apoptosis.

Although the direct consequence from Alternol-induced ROS accumulation was apoptotic cell death in cancer cells (18, 22, 23), multiple cellular signal kinases including MAPK, JNK and p38 were activated *via* a ROS-dependent mechanism (22). In addition, STAT3 activity was inhibited in Alternol-treatment osteosarcoma cells independent of ROS accumulation (22). The significance of these alterations requires further investigation.

## Attenuation of Cellular Autophagy Flux

Autophagy flux is an essential cellular machinery that regenerates nutrients by digesting damaged cellular proteins or organelles under energy stress condition (31). Cellular energy sensing AMPK and growth promoting AKT/mTOR pathways are the major modulators of autophagy flux (32). Early studies showed that Alternol treatment at 0.5 μM concentration induced a significant elevation of autophagy flux in benign prostate RWPE-1 but not in malignant prostate C4-2 cells (17). Autophagy response was evidenced by increased biosynthesis and processing of the LC3B protein, a key player in autophagy flux. Autophagy activation was associated with less cell death accompanied with increased AMPK activation in RWPE-1 cells. Consistently, inhibition of AMPK activity in RWPE-1 cells enhanced Alternol-induced cell death. These data indicate a pro-survival role of autophagy flux in benign cell after Alternol treatment.

On the other hand, a recent report showed that Alteronol at 1–2 μM concentrations induced autophagy response in malignant melanoma A375 and UACC62 cells, as evidenced by LC3B processing and cellular re-distribution, SQSTM1/p62 protein degradation and autophagic vacuole formation (26). Alternol-induced autophagy response was associated with reduced AKT/mTOR activation after Alteronol treatment. Autophagy inhibition with 3-MA or autophagy disruption by Bif-1 knockout enhanced Alteronol-induced cell death in A375 and UACC62 cells. In addition, Cladosporol was also reported to induce autophagy response *via* a ROS-dependent mechanism in breast cancer cells (11). These studies suggest that Alternol or Alteronol are capable of autophagy induction, leading to a protective effect on cell death.

## Inhibition of Cancer Cell Motility

Metastasis is the sole cause of cancer-related casualty and the metastatic potential is mainly defined by cancer cell motility (14). Matrix metalloproteinases (MMPs) are enzymes that degrade extracellular matrix and basement membrane, key factors in cancer metastasis (33). Alternol was shown to inhibit HepG2 cell migration and invasion, which was associated with reduced MMP-9 expression and a reversal of epithelial-to-mesenchymal transition (EMT) (19). Similarly, Alteronol was shown to inhibit cell invasion/migration *in vitro* and lung metastasis *in vivo* in murine melanoma B16F10 and B16F1 cells through a mechanism related to MMP2 reduction plus tissue inhibitor of metalloproteinases-2 (TIMP-2) induction (34). Consistently, in human melanoma A375 and UACC62 cells, Alteronol was also found to reduce cell invasion/migration *in vitro*, possibly through a TGFβ/Smad3 signal pathway-related epithelial-mesenchymal transition (EMT) mechanism (26). Not surprisingly, Cladosporol was found to reduce β-catenin protein level *via* a PPARγ-dependent proteasome degradation and to enhance E-cadherin gene expression, two strong regulators of cancer metastasis (9).

## Alteration of Cancer Cell Energy Metabolism

Metabolic reprogramming is a malignant hallmark and targeting metabolic pathway has been a hotspot in anti-cancer drug development (35, 36). To identify the protein targets, a recent report used a biotin labeled Alternol coupled with avidin beads to pull down cellular proteins that bond with Alternol (37). The eluted proteins were processed using Mass-Spectrometry approach and fourteen identified proteins were verified in western blot assays. Among them are four enzymes involved in the Krebs cycle, including the E2 component dihydrolipoyllysine-residue acetyltransferase (DLAT) of pyruvate dehydrogenase complex (PDHC), the E2 component dihydrolipoyllysine-residue succinyltransferase (DLST) of α-ketoglutarate dehydrogenase complex (KGDHC), fumarate hydratase (FH) and malate dehydrogenase-2 (MDH2). In prostate cancer cells, PDHC or KGDHC activities at the basal condition were significantly higher than that in benign prostate BPH1 cells, while Alternol treatment reduced PDHC and KGDHC activities in cancer cells to the levels close to that in BPH1 cells. Although FH and MDH2 activities were comparable among prostate cancer and benign cell lines at the basal condition, interestingly, Alternol enhanced their activities in prostate cancer cells but not in BPH1 cells. Further analysis using metabolomic approaches revealed that Krebs cycle intermediates, including citric acid, succinic acid, fumaric acid and malic acid, were much higher in malignant cells compared to benign cells under basal condition. Alternol treatment remarkably reduced the levels of malic acid, fumaric acid, and isocitric acid and mitochondrial respiration in prostate cancer cells. Consequently, mitochondrial respiration and ATP production were drastically reduced after Alternol treatment in prostate cancer PC-3 cells *in vitro* or in PC-3 cell-derived xenograft tissues but not in BPH1 cells or host liver tissues (37). These studies demonstrated that malignant cells posse a higher metabolic activity for energy production and that Alternol specifically interferes with the Krebs cycle enzymes, resulting in reduced ATP production and energy crisis in malignant cells and xenograft tissue.

## Conclusion

**FIGURE 4:**
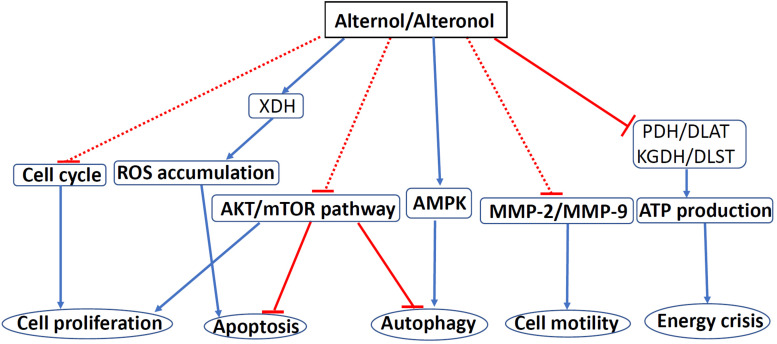
The mechanisms of Alternol/Alteronol-induced anti-cancer effects. Alternol and Alteronol reduce cell proliferation *via* cell cycle arrest, trigger apoptotic cell death *via* ROS accumulation and AKT/mTOR inactivation and attenuate cell motility by down-regulating MMP-2/9 expression. Alternol enhances autophagy flux *via* AMPK activation/AKT-mTOR inactivation and causes energy crisis by inhibiting Krebs’ cycle enzymes PDH/DLAT and KGDH/DLST complexes. Blue solid arrows denote a direct stimulating effect. Red solid lines denote a direct suppressive effect. Red dotted lines denote an indirect suppressive effect. Abbreviations: AMPK, AMP-activated protein kinase; DLAT, dihydrolipoyllysine-residue acetyltransferase; DLST, dihydrolipoyllysine-residue succinyltransferase; KGDH, a-ketoglutarate dehydrogenase; MMP, matrix metalloproteinase; mTOR, mammalian target of rapamycin; PDH, pyruvate dehydrogenase; ROS, reactive oxygen species; XDH, xanthine dehydrogenase.

Alternol and Alteronol are relatively new compounds with potent anti-cancer effects *via* multiple mechanisms, including cell cycle arrest, cell motility reduction, intrinsic apoptosis, ROS stress, and metabolic disruption (Figure 4 and Table 1). Most interestingly, Alternol was found to interact with four Krebs cycle enzymes, resulting in the disruption of ATP production and energy crisis specifically in cancer cells or xenograft tumors without affecting benign or host tissues. This malignant tissue selectivity provides a huge safety advantage over current clinical chemotherapy that targets all proliferating cells.

## Author Contributions

WL, JL, and BL wrote the draft. BL, JC, JHu, and JHo revised the manuscript. All authors contributed to the article and approved the submitted version.

## Conflict of Interest

JC was employed by the company SungenBio Inc. The remaining authors declare that the research was conducted in the absence of any commercial or financial relationships that could be construed as a potential conflict of interest.
